# Allometric conservatism in the evolution of bird beaks

**DOI:** 10.1002/evl3.267

**Published:** 2021-12-27

**Authors:** Louie M. K. Rombaut, Elliot J. R. Capp, Christopher R. Cooney, Emma C. Hughes, Zoë K. Varley, Gavin H. Thomas

**Affiliations:** ^1^ Department of Animal and Plant Sciences University of Sheffield Sheffield S10 2TN United Kingdom; ^2^ Department of Life Sciences Natural History Museum London London SW7 5BD United Kingdom; ^3^ Bird Group Department of Life Sciences The Natural History Museum Tring HP23 6AP United Kingdom

**Keywords:** Allometry, bird beaks, constraints, evolutionary conservatism

## Abstract

Evolution can involve periods of rapid divergent adaptation and expansion in the range of diversity, but evolution can also be relatively conservative over certain timescales due to functional, genetic‐developmental, and ecological constraints. One way in which evolution may be conservative is in terms of allometry, the scaling relationship between the traits of organisms and body size. Here, we investigate patterns of allometric conservatism in the evolution of bird beaks with beak size and body size data for a representative sample of over 5000 extant bird species within a phylogenetic framework. We identify clades in which the allometric relationship between beak size and body size has remained relatively conserved across species over millions to tens of millions of years. We find that allometric conservatism is nonetheless punctuated by occasional shifts in the slopes and intercepts of allometric relationships. A steady accumulation of such shifts through time has given rise to the tremendous diversity of beak size relative to body size across birds today. Our findings are consistent with the Simpsonian vision of macroevolution, with evolutionary conservatism being the rule but with occasional shifts to new adaptive zones.

**Impact Summary**

The traits of organisms do not evolve independently, but rather tend to evolve predictably in relation to each other. To what extent is diversity constrained in this way and over what timescales are such constraints broken to allow the range of life's diversity to expand? We address these fundamental questions about the tempo and mode of evolution with comparative data on beak size and body size of the world's birds. We find that the relationship between beak size and body size generally remains stable among sets of related species over millions of years. Such stability is broken over longer timescales, however, as shifts in the relationship between beak size and body size have accumulated steadily over time.

George Gaylord Simpson was an influential contributor to the theory of macroevolution (Simpson 1944, 1953). Simpson proposed that the majority of evolution takes place within “adaptive zones,” bounded regions of trait space characterized by relatively constrained rates of directional trait evolution, in which species evolve to partition sets of available niches. According to Simpson, this pattern of evolution is punctuated by occasional shifts to new adaptive zones triggered by ecological opportunity in new environments or following mass extinctions, or they may be spurred by the evolution of key innovations in functional traits. Taken together, this implies an overall pattern of generally conservative evolution, broken by occasional bursts of rapid directional evolution over long macroevolutionary or “megaevolutionary” timescales.

One way in which evolution may be conservative is in terms of allometry, a scaling relationship between the traits of organisms and body size following a power law of the form $y=Ax^{b}$ (Huxley 1932; Lande 1979). Allometry is a ubiquitous feature of biological variation, both within species and across species from micro‐ to macroevolutionary levels (Conner et al. 2014; Voje et al. 2014; Outomuro and Johansson 2017). Trait allometry across species emerges from constraints on evolution. These can be classified into genetic‐developmental, functional, and ecological constraints. When genetic‐developmental constraints are conserved across species, evolutionary allometry may emerge from allometry in the growth and development of the organism (Huxley 1932). Stabilizing selection on the function of traits can also help to maintain allometric conservatism in trait evolution (e.g., Skandalis et al. 2017). Ultimately, limits on ecological opportunity may prevent lineages from breaking free from conserved allometric relationships to adapt to new niches (e.g., Grant and Grant 2006).

As a consequence of genetic‐developmental, functional, and ecological constraints, allometric relationships may be conserved over timescales spanning millions to tens of millions of years (Voje et al. 2014; Houle et al. 2019). At one extreme is the possibility that lineages within major taxa evolve according to a strict Simpsonian model of evolution in which the allometric relationships between traits across different clades are strongly conserved through time and species in different clades rarely converge on the same trait combinations. In this scenario, variation between clades outweighs variation within clades. At the other extreme is the possibility that shifts in allometric relationships are so frequent that all lineages effectively evolve under the same loose allometric regime with extensive convergent evolution. Under this evolutionary scenario, variation within major clades can outweigh variation between clades. Observed evolutionary patterns may lie somewhere between these two qualitative extremes. At the scale of an entire class of organisms, measuring the extent and phylogenetic distribution of allometric conservatism is key to understanding the macroevolutionary dynamics that give rise to present day diversity.

The bird beak is an interesting anatomical trait on which to study evolutionary allometric conservatism. Bird beaks span a great range of sizes relative to body size, from the relatively long beaks of small hummingbirds to the relatively short beaks of ostriches. This diversity is closely associated with adaptation to different ecological niches (Pigot et al. 2020). In birds, there is strong allometry within certain clades between the shape and size of the beak (Bright et al. 2016, 2019; Navalón et al. 2020), and also between the size of the beak and body size (Van Den Elzen and Nemeschkal 2007; Shatkovska and Ghazali 2020). This pattern is scale dependent, however. Correlation between beak shape and size is relatively much weaker across major bird clades than it is within bird clades (Felice and Goswami 2018). This implies a breakdown in allometric conservatism on megaevolutionary timescales through multiple shifts in allometric relationships.

It remains unclear where on the bird phylogeny shifts in allometric relationships have taken place and what is the pattern of shifts through time that has ultimately produced the striking diversity of beak size in relation to body size we observe today across the world's birds. In this study, we aim to localize shifts in the allometric relationship between beak size and body size across the branches of the bird phylogeny. Using these inferred shifts, we test whether disparity in allometric relationships among bird clades expanded early in the history of crown birds with a subsequent deceleration, consistent with expectations under an adaptive radiation model in the wake of the ecological vacuum left by the K‐Pg mass extinction.

## Methods

### MORPHOLOGICAL DATA

We extracted bill centroid size measurements (mm) from landmarked three‐dimensional scans of the bills of museum specimens. These scans were previously obtained as part of a broader project and details of specimen selection, scanning, and landmarking can be found in Cooney et al. (2017). Typically, only one adult male individual was sampled per species. Centroid size is defined as the square root of the sum of squared Euclidean distances between each landmark and their centroid. Bill length, width, and depth were also measured for a majority of the same specimens and we found a very close relationship between these linear dimensions and bill centroid size in a multiple linear regression with the log‐linear dimensions being the predictors and log bill centroid size being the response (*R* ^2^ = 0.97).

We obtained body mass data (g) for males of each species from the species‐level medians reported by Myhrvold et al. (2015), as well as raw body mass data from museum records (www.vertnet.org). For the latter, we performed an automated error screening and removed 453 out of 28,355 anomalous records that were likely the result of human error. We then calculated species‐level averages by taking the mean of log‐transformed data. We computed a mean of the two sources for each species, weighted by sample size. The median total sample size per species was three individuals.

### PHYLOGENY

We downloaded a sample of 1000 “Hackett stage 2” trees from www.birdtree.org (Jetz et al. 2012), representing the posterior distribution of phylogenetic relationships among 9993 extant bird species based on molecular sequence data plus taxonomic imputation. We constructed a maximum clade credibility (MCC) tree from this sample of trees using TreeAnnotator (Bouckaert et al. 2019). Clades of the MCC tree were grafted onto corresponding nodes of the Prum et al. (2015) dated backbone phylogeny, as described in Cooney et al. (2017). The Prum et al. (2015) backbone phylogeny has posterior probability support for all but one of the nodes equal to 1.0. We mapped taxonomic labels for all other data to the species names in Jetz et al. (2012) with the aid of synonym tables from Avibase (www.avibase.org).

### SHIFTS IN ALLOMETRIC RELATIONSHIPS

To infer shifts in allometric relationships across the phylogeny, we used the rjMCMC method implemented in bayou (Uyeda and Harmon 2014; Uyeda et al. 2017). Bayou can fit models with multiple evolutionary regimes, each following an Ornstein‐Uhlenbeck (OU) process of trait evolution with a primary optimum parameterized by an allometric intercept *θ* and slope *β*. The relationship between bill centroid size (lnC) and body size (lnM) of species *j* is modeled as lnC*_j_* = **w** *_j_*_,_ *_α_* **θ** + *β_j_* lnM*_j_*, where **w** *_j_*_,_ *_α_* is a row vector of weights for each regime and **θ** is a column vector of all intercepts in the history of allometric regimes in which the lineage has evolved. Although shifts in allometry are modeled as discrete events, traits are assumed to evolve toward their new optima gradually at a rate proportional to *α*. The OU process is parameterized in terms of *α*, the evolutionary constraint parameter, and *σ* ^2^, the evolutionary rate parameter, but for each major clade in Table S1 we instead report the phylogenetic half‐life $\left(\frac{\ln(2)}{\alpha }\right)$, a measure of phylogenetic signal for traits evolving under an OU process, and the estimated stationary variance of the OU process $\left(\frac{\sigma^{2}}{2\alpha }\right)$, a measure of the expected residual variance around the allometric axis within regimes. These derived metrics are more readily interpretable. For further elaboration on the model and software implementation, see Uyeda et al. (2017).

We note that a Brownian motion process (BM) is a special case of an OU process with *α* = 0. We therefore did not fit a separate multi‐regime BM model to our data. Although intraspecific error can erode phylogenetic signal in data generated under a BM process and give the appearance that species have evolved under an OU process (Cooper et al. 2016), we find that our estimates of phylogenetic half‐life are robust to reasonable assumptions about intraspecific error (see below).

Because an analysis on our full dataset of 5083 species proved to be computationally intractable in any reasonable time frame, we split our data into 18 clades of up to 800 species (see Table S1) and ran analyses on these clades in parallel. A limitation of this approach is that we cannot explicitly test for shifts at the base of these clades. Instead, we compare the 95%HPD for the parameters at the root of each clade to the parameters inferred under a global model that reflects an average allometric relationship (see below).

For each clade‐level analysis, we set a Poisson prior distribution on the number of allometric shifts with a *λ* parameter of 2% of the number of species in that clade rounded up to the nearest integer. We placed a uniform prior on the probability of a shift over all branches on the phylogeny and also on the locations of shifts along branches. We set half‐Cauchy prior distributions on *α* and *σ* ^2^ with a scale parameter of 0.1. We set weakly informative normal priors on the slopes (mean = 0.33, SD = 0.5) and intercepts (mean = 2, SD = 0.5) of each regime. The slope prior is based on an isometric relationship between bill size and body mass. Because bill centroid size has dimensions of length while body mass may be assumed to be proportional to volume that has dimensions of length cubed, an allometric slope of ∼1/3 is consistent with isometry, meaning that relative bill size stays the same as body size increases across species. A slope greater than 1/3 implies positive allometry, meaning that bills become relatively larger as body size increases across species. A slope less than 1/3 implies negative allometry, meaning that bills become relatively smaller as body size increases across species. If slope is held constant, the allometric intercept reflects the relative size of the bill for a given body size.

For each clade‐level analysis, we ran replicate MCMC chains in parallel, sampling every 200 generations until convergence was reached with a burnin proportion of 50%. For Coraciimorphae and Aequorlitornithes, we ran four replicate chains for 100 million generations each. We ran four replicate chains for 60 million generations each for Accipitriformes, Columbaves, Galliformes, Nectariniidae, Passeroidea, and Sylvioidea. For the remaining clades, we ran duplicate chains for 30 million generations each. We also fit a model with a single allometric regime across all birds. Duplicate chains for this model were run for 2 million generations with a burnin proportion of 20%.

Parameters monitored for convergence include the likelihood, the number of inferred shifts and their locations, as well as *α* and *σ* ^2^. To conclude that the chains had reached convergence, we required an effective sample size greater than 200 for each parameter in each chain and a value of Gelman and Rubin's *R* diagnostic below 1.05 (Gelman and Rubin 1992). For some clades, the traces of the R statistic for *α* and *σ* ^2^ followed a somewhat erratic pattern. We speculate that this may be due to the identifiability issue inherent in jointly estimating *α* and *σ* ^2^ and/or a consequence of the nature of the half‐Cauchy prior distributions on these parameters generating large outliers. In any case, we found that the posterior distributions for *α* and *σ* ^2^ were identical between chains in the bulk of the distribution and only differed in terms of outliers in the tails.

To identify the locations of shifts, we visualized the distribution of posterior probability support for the presence of shifts across the branches of the phylogeny. Because support for a shift may sometimes be “smeared” across a set of adjacent branches, rather than relying on a posterior probability cutoff per branch to infer shifts we first identified regions with elevated support for the presence of a shift and then we located shifts on branches with the maximum posterior probability for a shift. Where the posterior probability for a shift was similar between adjacent branches, preference was given to the most rootward branch encompassing the possibility that the shift occurred along a descendent branch. We used the posterior distribution on the number of shifts as a guide to how many shifts there were to identify in each clade. Using these fixed shift locations, we estimated the allometric slope and intercept across evolutionary regimes by rerunning bayou chains in duplicate for 20 million generations for each of the 18 major clades. We assessed convergence as per the first set of analyses.

### MEASUREMENT ERROR AND SENSITIVITY ANALYSES

We performed sensitivity analyses to gauge the impact of intraspecific variation in bill size and body size on our inferences (Ives et al. 2007; Silvestro et al. 2015). Error in predictor variables can cause attenuation bias in regression slopes (Hansen and Bartoszek 2012). As an indication of the impact this has on our slope estimates, we calculated the reliability ratio for ordinary least squares regression using an ANOVA on body mass data for each of the allometric regimes we identified with more than 10 species. Dividing the estimated slope by the reliability ratio yields the true slope corrected for attenuation. We found that the median reliability ratio was 0.99, and we therefore conclude that our results are generally robust to slope attenuation bias. Intraspecific variation in bill size may inflate our estimates of the allometric residual variance within regimes. We do not have enough repeat measurements within species to estimate intraspecific variation in bill centroid size measurements from our own data. We therefore turned to published data and found that the intraspecific coefficient of variation in bill size ranges between ∼3% and 7% across bird species (Montoya et al. 2018; Rodrigues et al. 2019; Cardona‐Salazar et al. 2020; Kennedy et al. 2020; Tsai et al. 2020). Bayou allows the user to specify fixed measurement error estimates to be taken into account in the inference of parameters. We reran bayou chains on the Muscicapoidea, Falconiformes, Coraciimorphae, and Sylvioidea clades with estimates of intraspecific coefficients of variation of 3%, 5%, and 7%. These clades were chosen as examples because they reflect a range of estimated allometric conservatism. We found that when accounting for a range of possible intraspecific variation, our estimates of stationary variance for these clades are largely congruent with what we estimated in our main analyses that assumed zero measurement error (table s2).

### TEMPORAL TRENDS IN ALLOMETRIC SHIFTS AND THE DIVERSITY OF ALLOMETRIC REGIMES

We used our estimates of shifts in allometric slope and intercept across the phylogeny to test for trends in the accumulation of allometric disparity (i.e., variation in slopes and intercepts of allometric relationships) through time. For each branch on which we inferred a shift, we took the midpoint of the branch as the point in time when the shift occurred. We restricted our analysis to regimes containing at least 10 extant species. The root regimes for each of our 18 clades were included only if the 95% HPD intervals for either slope or intercept did not overlap with the slope and intercept estimates of the global allometric model across all bird species. We excluded the Strisores root regime as an outlier because we have reason to believe that the model is misspecified for this regime on account of a major difference in bill shape between potoos, oilbirds, and nightjars and the most basal clade of hummingbirds. We first used principal component analysis to generate a space of slopes and intercepts across all regimes. We then inferred an empirical disparity through time curve using the R package dispRity (Guillerme 2018) by calculating the cumulative sum of Euclidean distances from the centroid of the slope‐intercept data to each slope‐intercept coordinate in principal component space over time. We next generated a null distribution of 1000 disparity through time curves. If shifts have accumulated at a constant rate through time, the probability of recovering a shift in a given time slice should depend on the number of branches in that time slice, with a greater probability of recovering a more recent shift than an ancient one. We therefore pruned the phylogeny to branches subtending at least 10 species and sampled random time slices for the occurrence of our observed shifts in proportion to the number of branches of the phylogeny present in those time slices. We tested for departures from the null distribution of curves in our empirical disparity through time curve using the rank‐envelope test (Murrell 2018).

## Results and Discussion

### ALLOMETRIC DIVERSITY

**Figure 1:**
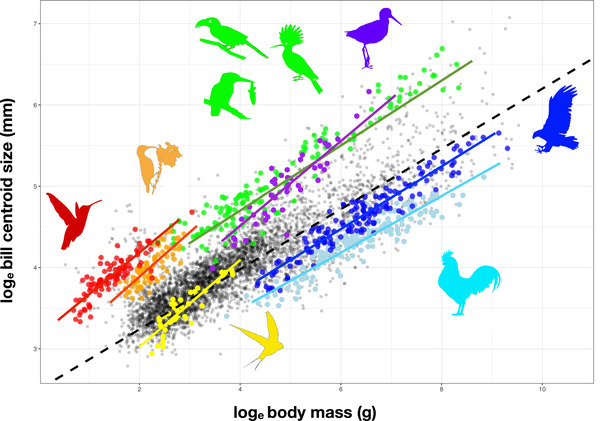
Allometric relationships between beak size and body size illustrated for several clades. Bird silhouettes were obtained from phylopics.org under a creative commons license.

**Figure 2:**
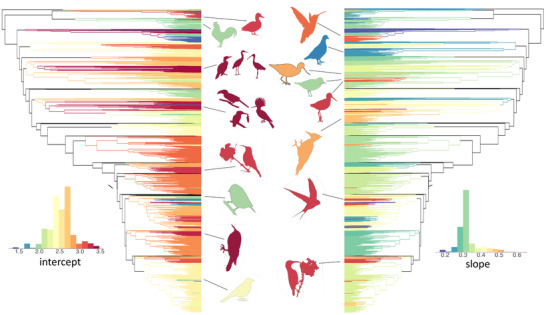
The phylogenetic distribution of allometric intercepts and slopes. Histogram insets show the frequency distributions of parameters across branches of the phylogeny. Bird silhouettes represent the following clades: (left‐hand side from top to bottom) ducks and geese; fowls; cormorants, herons, ibises, etc.; hornbills, hoopoes, kingfishers, etc.; honeyeaters; tits; treecreepers, etc.; finches, etc.; (right‐hand side from top to bottom) hummingbirds; doves, etc.; oystercatchers and stilts; stints and turnstones; sandpipers; woodpeckers; swallows; sunbirds. Further details on clades can be found in Table S1. Bird silhouettes were obtained from phylopics.org under a creative commons license.

We identified 53 statistically supported shifts in allometric relationships across the bird phylogeny that subtend clades with at least two representative species (Figs. 1 and 2; Table S1). We also identified many additional shifts on branches supporting a single species (Table S3). Although some of these are likely candidates for lineages that have experienced an allometric shift, such as the sword‐billed hummingbird *Ensifera ensifera*, the scythebill *Campylorhamphus trochilirostris*, and the palm cockatoo *Probosciger aterrimus*, other outliers more likely reflect errors in bill size and/or body size data or phylogenetic placement. Although some shifts subtend large and ancient clades with upward of 100 representative species, the majority of shifts subtend young clades with relatively few species. For the 53 shifts we inferred, the median number of sampled species in clades subtended by a shift is 14 and the median shift age is 14 Myr. Some shifts are nested within clades that have themselves shifted from the ancestral allometric relationship within their clade.

We find examples of several different patterns in the evolution of bill size relative to body size in birds. We find cases of parallelism in allometric relationships consistent with ecological convergence between clades. We find such parallelism between the sandpipers and a clade comprising the stilts, avocets, and oystercatchers, and also between hummingbirds and sunbirds. The most common example of convergence we find is among nectar‐feeding birds independently acquiring relatively long bills to extract nectar from flowers. Allometric shifts are usually associated with the acquisition of a specific ecological niche that remains conserved across species, but we also find cases where there has been extensive ecological divergence despite relative conservatism in the allometric relationship between bill size and body size. The most notable examples are the Pelecaniformes (gannets, cormorants, herons, etc.) and the core Coraciimorphae (hornbills, bee eaters, kingfishers, etc.). Finally, we also find examples of evolutionary reversals from extremes of relative bill size to bill morphology more typical of birds as a whole. Notable examples are the sunbird genus *Anthreptes*, the honeyguides among the Piciformes (woodpeckers, toucans, and barbets), and the stints and turnstones among the sandpipers.

The majority of bird lineages evolve around a nearly isometric slope in the relationship between beak size and body size (Fig. 2, histogram insets), so that differences in relative beak size between clades are usually mostly attributable to shifts in intercept. This distribution of slopes is skewed, however, with some clades having strongly positive allometry accounting for large beak size relative to body size in some species. It may be hypothesized that this diversity reflects differences in the underlying developmental trajectories producing variation in adult morphologies. Shifts in intercept may reflect changes in the relative rate of growth of the beak early in development, whereas a steep allometric slope across species may reflect a rapid rate of beak growth relative to body size that is sustained during postnatal development and conserved across closely related species. These hypotheses remain to be tested with comparative ontogenetic data.

### ALLOMETRIC CONSERVATISM

The degree of conservatism within allometric regimes can be quantified using our estimates of stationary variance under an OU model, which predicts the expected residual variance around the slope of the allometric relationship. Our estimates of stationary variance ($\sigma^{2}$) can be expressed as coefficients of variation (cv) using the transformation $\mathrm{cv}=\sqrt{e^{\sigma^{2}}-1}\;$. Across the 18 major bird clades we analysed, the median coefficient of variation in bill size for a given body size is 15%. This can be contrasted with a coefficient of variation of 34% under a model assuming all bird species evolve under a single allometric regime. As a further point of comparison, the maximum range in bill size for species of the same body size is roughly between 35% below and 160% above the expected mean, corresponding to the difference in bill size between two species of the same body size, the piping plover *Charadrius melodus* and the rufous‐lored kingfisher *Todiramphus winchelli*. There is thus evidence for strong conservatism within allometric regimes.

### TEMPORAL TRENDS IN ALLOMETRIC SHIFTS AND THE DIVERSITY OF ALLOMETRIC REGIMES

**Figure 3:**
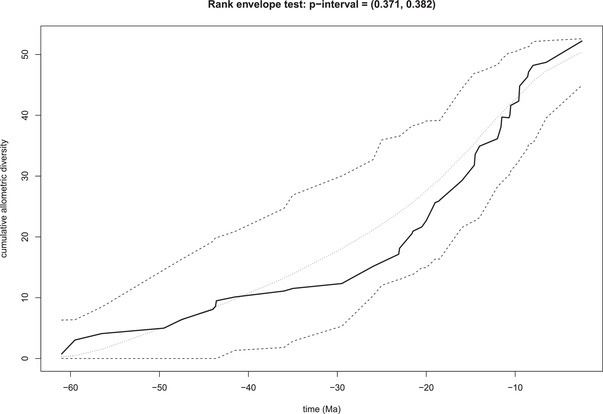
The accumulation of allometric diversity through time measured as the cumulative sum of Euclidean distances of slope‐intercept coordinates to the centroid of slope‐intercept space. The solid line is the empirical curve of accumulation and the dotted lines represent the upper and lower 95% simulated confidence limits under the null model.

**Figure 4:**
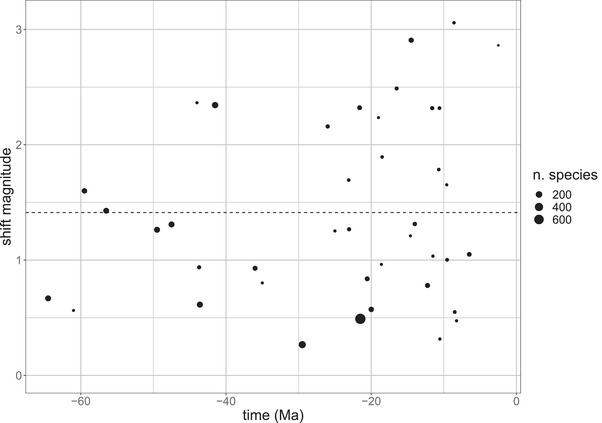
The magnitude of inferred shifts through time measured as the Euclidean distance in slope‐intercept space to ancestral regimes. Each point represents a distinct allometric regime and point size is proportional to number of species presently within that regime. The slope of the relationship is nonsignificant (*P* > 0.05).

Although the range of bird bill size in relation to body size may have expanded early, allometric diversity across clades has accumulated at a steady rate through time with no evidence that the rate of shifts or the average magnitude of shifts has changed through time (Figs. 3 and 4). Rather than an early burst of divergence followed by stasis as species pack ever more tightly within existing sets of niches, the pattern we recover suggests that bird lineages have continued to colonize new adaptive zones via allometric shifts up to the present day. The observed cumulative disparity through time curve bends upward toward the present, consistent with the null expectation that more recent shifts have a greater probability of being recovered than more ancient shifts, analogous to the pull of the present in lineage through time plots (Nee et al. 1994).

The number of distinct allometric regimes we can recover is a question of power to detect shifts in allometry. Our power to resolve allometric regimes is limited by the length of time that lineages have had to evolve within distinct regimes, the magnitude of difference in slope and intercept between regimes, and the degree of allometric conservatism within regimes (Ho and Ané 2014; Uyeda and Harmon 2014). Because we use comparative data from living taxa, an important limitation of our work is that we cannot detect ancient shifts in allometry in lineages whose descendants have all become extinct. We are limited to reconstructing the history of allometric shifts that have given rise to the present diversity of bird beaks, which may or may not be representative of the complete history of bird beak evolution in species extinct and extant (Mitchell 2015).

### ALLOMETRIC CONSERVATISM: ECOLOGICAL OPPORTUNITY VERSUS GENETIC‐DEVELOPMENTAL CONSTRAINTS

What are the causes of allometric conservatism over macroevolutionary timescales? Genetic‐developmental constraints could be an important driver of conservatism from micro‐ to macroevolutionary timescales. Potential genetic‐developmental constraints on variability include linkage disequilibrium between genes, pleiotropy of genes and new mutations, as well as epistatic interactions promoting developmental canalization by buffering the developing organism from environmental and mutational noise (Walsh and Blows 2009; Svensson et al. 2021). Maintaining robustness in the growth and development of the phenotype is likely a target of stringent selection. Rather than being an impediment to adaptation, however, genetic‐developmental constraints may in fact facilitate adaptative evolution if the direction of selection is usually aligned with genetic lines of least resistance (Schluter 1996). Theoretically, a certain degree of pleiotropy is also optimal for evolvability because it presents a bigger target for mutations on which selection can act (Hansen 2003). For these reasons, genetic‐developmental constraints may remain conserved across species over macroevolutionary timescales. Although progress has been made in our understanding of the genetic‐developmental basis of variation in bird beak size and shape (Grant et al. 2006; Mallarino et al. 2011, 2012), more research is necessary to understand the mechanistic basis of constraints on the independent evolution of the beak (Fritz et al. 2014).

Genetic‐developmental constraints alone cannot be a sufficient explanation for macroevolutionary conservatism. We know of several examples of rapid evolution suggesting that genetic‐developmental constraints may be readily broken given the right selection pressures. For instance, under artificial selection domesticated pigeons have evolved a striking diversity of beak size and shape (Young et al. 2017). The island radiations of Darwin's finches, Hawaiian honeycreepers, and Madagascan vangas are further examples of rapid adaptive evolution in response to selection (Lovette et al. 2002; Reddy et al. 2012; Navalón et al. 2020). In this study, we have identified several other lineages that have diverged rapidly from their ancestors in the recent past, against a background of allometric conservatism. It is likely that macroevolutionary conservatism is maintained by an interaction between genetic‐developmental constraints and limits on ecological opportunity for shifts to new adaptive zones.

## AUTHOR CONTRIBUTIONS

LR and GT conceived and designed the study. EC, CC, EH, and ZV collected the data. LR analyzed the data and drafted the manuscript. All authors reviewed the manuscript.

## CONFLICT OF INTEREST

The authors declare no conflict of interest.

## DATA ARCHIVING

Data used in this study can be accessed on figshare: https://figshare.com/articles/dataset/body_mass_and_beak_size_data_for_the_world_s_birds/16556145.

## Supporting information

Supplementary information

Supplementary information

Supplementary information
