# Supplementary material for: Allometric conservatism in the evolution of bird beaks
Source: Evol Lett. 2021 Dec 27;6(1):83–91. doi: 10.1002/evl3.267 (PMC8802239; doi:10.1002/evl3.267)
Supplement: Supplementary file 1 — Supplementary information [file EVL3-6-83-s002.pdf]

Table 1. Allometric Shifts and Parameter Estimates

ShiftID	Clade	n=	slope (95%HPD)	intercept (95%HPD)	phylogenetic half-life (Myr) (95%HPD)	stationary variance (cv%) (95% HPD)	shift age ~(Ma)
Palaeognathae (ratites & tinamous)					0.17 (0.05-17)	8% (5.8-12.1)	
1.0	root	13	0.31 (0.28-0.33)	2.50 (2.32-2.69)			50-72
1.1	kiwis (*Apteryx sp.*)	4	-	-			4-26
1.2	select tinamous (*Crypturellus sp.*)	5	-	-			~23
Anseriformes (ducks & geese)					1.4 (0.8-3.3)	15% (12.4-17.8)	
2.0	root	89	0.32 (0.27-0.37)	2.79 (2.45-3.12)			46-54
2.1	swans & geese (*Anserinae*)	19	0.44 (0.37-0.52)	1.49 (0.82-2.12)			~9
2.2	teals & shovelers (*Spatula sp.*)	10	0.50 (0.38-0.63)	1.71 (0.80-2.60)			~3
2.3	sheldgeese (*Chloephaga sp.*)	4	-	-			~3
Galliformes (fowls)					0.6 (0.3-2.0)	14% (12.4-15.5)	
3.0	root	150	0.34 (0.32-0.37)	2.14 (1.98-2.30)			45-54
3.1	guans & curassows (*Craciidae*)	23	0.28 (0.21-0.35)	2.76 (2.23-3.26)			9-37
3.2	grouse (*Tetraoninae*)	15	0.30 (0.22-0.38)	2.21 (1.65-2.75)			~9
Strisores (nightjars, swifts & hummingbirds)					45 (26-165)	42% (32.1-83.0)	
4.0	root	51	0.23 (0.13-0.33)	3.36 (2.86-3.81)			64-67
4.1	coquettes & brilliants	51	0.34 (0.21-0.48)	2.12 (1.23-2.97)			~15
4.2	emeralds, mountain gems & bees	96	0.51 (0.44-0.59)	1.72 (0.86-2.58)			~15
4.3	swifts (*Apodidae*)	14	0.23 (0.05-0.42)	1.86 (0.96-2.75)			41-47
Columbaves (cuckoos, bustards & doves)					6.2 (4.3-10.4)	15% (13.7-17.2)	
5.0	root	146	0.31 (0.28-0.34)	2.44 (2.28-2.62)			62-67
5.1	cuckoos (*Cuculidae*)	81	0.37 (0.32-0.42)	2.70 (2.47-2.93)			~36
5.2	sandgrouse (*Pteroclidae*)	13	0.24 (0.10-0.37)	2.35 (1.60-3.14)			17-53
Gruiformes (rails, crakes & cranes)					8.6 (4.6-33)	28% (22.3-40.2)	
6.0	root	74	0.34 (0.30-0.39)	2.60 (2.31-2.89)			40-65
Aequorlitornithes (gulls, sandpipers, herons etc.)					20 (15-31)	31% (26.8-36.5)	
7.0	root	266	0.35 (0.32-0.38)	2.56 (2.36-2.77)			~64
7.1	stilts, avocets & oystercatchers (*Recurvirostridae + Haematopidae*)	15	0.46 (0.35-0.56)	2.51 (1.59-3.43)			22-28
7.2	curlews, godwits & other sandpipers (*Scolopacidae*)	44	0.52 (0.44-0.60)	2.21 (1.48-2.89)			22-30
7.3	stints & turnstones (*Eroliinae*)	22	0.36 (0.25-0.47)	2.49 (1.62-3.34)			17-20
7.4	gannets, cormorants & herons (*Pelecaniformes*)	109	0.37 (0.32-0.41)	3.27 (2.85-3.71)			~60
Accipitriformes (vultures, hawks & eagles)					0.2 (0.08-1.2)	13% (11.9-15.1)	
8.0	root	149	0.39 (0.37-0.41)	2.15 (2.02-2.29)			39-56
8.1	New World vultures (*Cathartidae*)	4	-	-			6-56
8.2	serpent eagles (*Spilornis sp.*)	5	-	-			~5

Table 1. Allometric Shifts and Parameter Estimates

ShiftID	Clade	n=	slope (95%HPD)	intercept (95%HPD)	phylogenetic half-life (Myr) (95%HPD)	stationary variance (cv%) (95% HPD)	shift age ~(Ma)
	Coraciimorphae (hornbills, bee eaters, kingfishers etc.)				17 (13-27)	20% (17.5-23.6)	
9.0	root (owls, trogons & mousebirds)	45	0.32 (0.24-0.39)	2.57 (2.17-2.94)			~61
9.1	core coraciimorphae (hornbills, bee eaters, kingfishers etc.)	137	0.39 (0.35-0.43)	3.18 (2.97-3.43)			~57
9.2	rollers (*Coraciidae*)	8	-	-			15-43
9.3	jacamars (*Galbulidae*)	8	-	-			22-34
9.4	woodpeckers & barbets (*Piciformes*)	182	0.46 (0.42-0.49)	2.30 (1.97-2.54)			34-49
9.5	honeyguides (*Indicatoridae*)	13	0.39 (0.32-0.45)	2.07 (1.50-2.60)			14-23
9.6	toucans (*Ramphastidae*)	13	0.57 (0.49-0.65)	2.32 (1.42-3.21)			13-16
	Falconiformes (falcons)				0.1 (0.04-1.1)	10% (7.9-12.2)	
10.0	root	39	0.34 (0.30-0.38)	2.27 (2.04-2.49)			29-58
10.1	caracaras (*Caracarinae*)	8	-	-			8-17
	Psittaciformes (parrots)				4.1 (2.7-8.0)	16% (13.6-19.1)	
11.0	root	161	0.36 (0.33-0.39)	2.40 (2.24-2.57)			32-55
	Suboscines (ovenbirds, cotingas & manakins etc.)				10 (7.6-14)	18% (16.8-21.0)	
12.0	root	424	0.35 (0.32-0.38)	2.67 (2.55-2.79)			41-46
12.1	woodcreepers (*Dendrocolaptinae*)	23	0.49 (0.38-0.60)	2.28 (1.57-2.97)			9-12
	Meliphagoidea (pardalotes, fairy wrens & honeyeaters etc.)				8.7 (5.4-19)	19% (16.8-24.6)	
13.0	root	70	0.33 (0.28-0.39)	2.59 (2.37-2.79)			39-46
13.1	honeyeaters (*Meliphagidae*)	107	0.37 (0.32-0.42)	2.85 (2.59-3.12)			18-22
	Corvoidea (crows, cuckooshrikes & birds of paradise etc.)				7.8 (5.7-11.7)	17% (15.6-19.6)	
14.0	root	338	0.36 (0.33-0.39)	2.66 (2.54-2.78)			~31
14.1	longrunners (*Orthonychidae*)	3	-	-			8-24
14.2	longbills (*Toxorhamphus sp.*)	2	-	-			3-11
14.3	riflebirds (*Ptiloris sp.*)	5	-	-			~5
14.4	*Drepanornis sp.*	2	-	-			~5
14.5	*Epimachus sp.*	2	-	-			~4
	Sylvoidea (tits, larks & warblers etc.)				0.11 (0.04-0.5)	11% (10.5-11.8)	
15.0	root	262	0.37 (0.35-0.39)	2.58 (2.52-2.63)			~30
15.1	tits & chickadees (*Paridae*)	40	0.43 (0.35-0.52)	2.15 (1.93-2.38)			21-25
15.2	larks (*Alaudidae*)	36	0.55 (0.46-0.63)	1.98 (1.69-2.27)			~16
15.3	select larks (*Galerida sp.;Spizocorys sp.* etc.)	27	0.37 (0.27-0.48)	2.46 (2.08-2.80)			9-12
15.4	acrocephalid warblers (*Acrocephalidae*)	28	0.42 (0.32-0.52)	2.58 (2.31-2.85)			~9
15.5	prinias, cisticolas & apalises (*Cisticolidae*)	87	0.32 (0.26-0.38)	2.80 (2.65-2.94)			~13
15.6	tailorbirds (*Orthotomus sp.*)	5	-	-			~8
15.7	crombecs & longbills (*Macrosphenidae*)	13	0.22 (0.11-0.33)	3.18 (2.88-3.49)			~20
15.8	swallows & martins (*Hirundinidae*)	41	0.53 (0.45-0.60)	1.98 (1.76-2.20)			10-13
15.9	select bulbuls (*Phyllastrephus sp.*)	16	0.39 (0.23-0.53)	2.67 (2.20-3.20)			9-12
15.10	old world babblers (*Timaliidae*)	14	0.51 (0.41-0.61)	2.34 (2.04-2.66)			8-11
15.11	jungle babblers (*Pellorneidae*)	29	0.29 (0.20-0.39)	2.94 (2.61-3.27)			~10
15.12	bushtits & long-tailed tit (*Aegithalidae*)	5	-	-			10-14

Table 1. Allometric Shifts and Parameter Estimates

ShiftID	Clade	n=	slope (95%HPD)	intercept (95%HPD)	phylogenetic half-life (Myr) (95%HPD)	stationary variance (cv%) (95% HPD)	shift age ~Ma)
Muscicapoidea (flycatchers, treecreepers & wrens etc.)					4.6 (3.4-6.7)	15% (14.1-16.8)	
16.0	root	347	0.34 (0.31-0.37)	2.64 (2.52-2.75)			~27
16.1	nuthatches, treecreepers & wrens (*Certhioidea*)	76	0.34 (0.26-0.41)	2.97 (2.74-3.21)			18-23
Nectarinoidea (leafbirds, flowerpeckers & sunbirds)					0.08 (0.03-0.7)	13% (11.0-14.5)	
17.0	root	21	0.40 (0.33-0.46)	2.53 (2.35-2.72)			24-27
17.1	sunbirds (*Nectariniidae*)	72	0.56 (0.47-0.66)	2.74 (2.53-2.94)			20-23
17.2	select sunbirds (*Anthreptes sp*.)	16	0.47 (0.27-0.68)	2.62 (2.15-3.07)			~12
17.3	spiderhunters (*Arachnothera sp.*)	4	-	-			19-22
17.4	sugarbirds (*Promerops sp.*)	2	-	-			6-15
Passeroidea (finches, sparrows & pipits)					4.1 (3.4-5.0)	15% (14.3-16.5)	
18.0	root	736	0.36 (0.34-0.39)	2.45 (2.37-2.54)			20-23
18.1	icterids (*Icteridae*)	66	0.45 (0.39-0.51)	2.31 (1.94-2.68)			~7
18.2	Hawaiian honeycreepers (*Drepanidinae*)	8	-	-			~2
18.3	grosbeaks (*Mycerobas sp.; Eophona sp*. etc.)	7	-	-			8-11
18.4	New World honeycreepers (*Cyanerpes sp*.)	3	-	-			~3
