# Supplementary material for: Allometric conservatism in the evolution of bird beaks
Source: Evol Lett. 2021 Dec 27;6(1):83–91. doi: 10.1002/evl3.267 (PMC8802239; doi:10.1002/evl3.267)
Supplement: Supplementary file 2 — Supplementary information [file EVL3-6-83-s001.pdf]

**Table S2. Intraspecific Error Sensitivity Analyses (median coefficient of variation of stationary OU variance and 95%HPD).**

| Clade | 3% | 5% | 7% |
| --- | --- | --- | --- |
| Muscicapoidea | 15.1 (13.8-17) | 14.9 (13.3-17.7) | 14.8 (12.6-20.3) |
| Falconiformes | 9.1 (7.2-11.8) | 8.1 (6-11.1) | 6.3 (3.1-9.8) |
| Coraciimorphae | 20.2 (17.3-25.4) | 21.1 (17.2-30.6) | 22.5 (17-92.2) |
| Sylvoidea | 10.7 (10.1-11.5) | 10.7 (10.1-11.5) | 9.2 (8-12) |
