# Supplementary material for: Allometric conservatism in the evolution of bird beaks
Source: Evol Lett. 2021 Dec 27;6(1):83–91. doi: 10.1002/evl3.267 (PMC8802239; doi:10.1002/evl3.267)
Supplement: Supplementary file 3 — Supplementary information [file EVL3-6-83-s003.pdf]

S3 Outlier Taxa

Cyanochen cyanoptera
Malacorhynchus membranaceus
Pauxi pauxi
Macrodipteryx longipennis
Ensifera ensifera
Ramphomicron microrhynchum
Oreonympha nobilis
Rhodopis vesper
Chalcostigma heteropogon
Myrtis fanny
Musophaga violacea
Cacomantis merulinus
Monias benschi
Ptilinopus perousi
Larus bulleri
Sterna vittata
Larus glaucescens
Ixobrychus sinensis
Ixobrychus minutus
Chondrohierax uncinatus
Strix aluco
Trogon viridis
Trogon chionurus
Aceros cassidix
Bycanistes fistulator
Indicator xanthonotus
Sphyrapicus ruber
Dendrocopos cathpharius
Dendrocopos leucotos
Dendrocopos obsoletus
Piculus flavigula
Lybius rolleti
Calyptorhynchus funereus
Probosciger aterrimus
Drymornis bridgesii
Campylorhamphus trochilirostris
Manorina melanocephala
Urolestes melanoleucus
Lophorina superba
Seleucidis melanoleucus
Muscicapa ussheri
Ficedula mugimaki
Cyornis superbus
Panurus biarmicus
Zosterops albogularis
Foudia eminentissima
Ploceus temporalis
Loxioides bailleui
Serinus syriacus
Serinus mennelli
Serinus buchanani
Plectrophenax nivalis

Arremon aurantiirostris
Arremon crassirostris
Nesospiza wilkinsi
Sporophila bouvronides
